# Editorial: Extracellular matrix in development and disorders of the nervous system

**DOI:** 10.3389/fcell.2023.1153484

**Published:** 2023-02-13

**Authors:** Igor Jakovcevski, Pavle R. Andjus, Eckart Förster

**Affiliations:** ^1^ Institut für Anatomie und Klinische Morphologie, Universität Witten/Herdecke, Witten, Germany; ^2^ Center for Laser Microscopy, Institute for Physiology and Biochemistry “Jean Giaja”, Faculty of Biology, University of Belgrade, Belgrade, Serbia; ^3^ Department of Neuroanatomy and Molecular Brain Research, Institute of Anatomy, Ruhr-Universität Bochum, Bochum, Germany

**Keywords:** extracellular matrix (ECM), nervous system, regeneration, perineuronal nets (PNNs), synapses

The extracellular matrix (ECM) plays important roles in development, function and repair of the damaged nervous system. It is composed of numerous molecules, including proteins and proteoglycans that are orchestrate cellular behavior during development, physiological function and plasticity in the mature nervous system, as well as repair of neural cells after damage. By playing an important role in synaptogenesis and stabilization of synapses, the ECM influences synaptic activity and plasticity of neuronal circuitries. The ECM does not only contain functionally important molecules, but is also a intercellular medium through which cell to cell signaling occurs, regulating many processes between cells within the nervous system. Of particular interest are so-called perineuronal nets, an ECM component which forms around some neurons in the central nervous system (CNS), stabilizing their synapses and playing important roles in plasticity. Increasing knowledge about the roles of the ECM and its components in CNS development and disease is important not only from a biological viewpoint, but also from the therapeutic perspective. Activation of signaling cascades underlying reorganization of the extracellular matrix and stimulation of neuronal plasticity is an experimentally achievable goal and appears as a promising approach for the treatment of acute and chronic neurodegenerative disorders. Recent data suggest that ECM matrix proteases not only modify cell recognition molecules on the surface of the neuronal plasma membrane, but also modulate synaptogenesis and plasticity during development and neurorepair (Dityatev et al., 2010; Bijelic et al.).

The main aim of this Research Topic, which spanned almost over the period of the year 2022, was to cover promising and novel research trends on ECM molecules and their roles in neurodevelopment, neurodegeneration and neuroregeneration.

Several studies in this Research Topic address molecular mechanisms of axonal pathfinding, one of the essential events during development and in neuronal regeneration (Loers and Schachner, 2007; Mannes and Schachner, 2007). Guo et al. established the role of cadherin 12 in promoting axonal extension *in vitro* and *in vivo* and proposed that this effect was mediated through the PKA/Rac1/Cdc42 pathway. This work exemplifies the successful combination of molecular biology, cell culture and *in vivo* experiments. Another study, combining *in vitro*—, *in silico* —and *in vivo* approaches, shows the roles of olfactorin, an ECM protein expressed in the mouse olfactory region along the migratory route of hypothalamic gonadotropin-releasing hormone (GnRH) neurons (Di Schiavi et al.). The authors show that olfactorin exerts a strong chemoattractive effect on GnRH neurons through the activation of the FGF-receptor and MAP-kinase pathways; therefore, the lack of olfactorin likely contributes to the development of the Kallmann’s syndrome (a condition that causes hypogonadotropic hypogonadism).

Moving deeper into translational research, Kulesskaya et al. studied possibilities to overcome the inhibitory effects of chondroitin sulfate proteoglycans (CSPGs) for axonal regeneration in the CNS by applying protamine, a cognate inhibitor of heparin used in cardiovascular surgery (Kaneda et al., 2005). They demonstrated that protamine blocks the inhibitory effects of CSPGs on axonal outgrowth in cultured neurons *in vitro*, and *in vivo* in the rodent model of spinal cord injury. Moreover, as the full-length form of protamine is neurotoxic, they successfully implemented a 14 amino acid peptide that was found to be as active as protamine, but, in contrast, has no toxic adverse effects. Further research to explore regeneration upon injury, analyzed in detail the effects of tenascin C (TnC), known to improve regeneration after spinal cord injury (Chen et al., 2010), on the astrocytes and microglia in injury models *in vitro* (Bijelic et al.) The authors show versatile functions of TnC and in particular of its FnD domain after injury, such as contributing to ongoing inflammation in the injured region. The FnD fragment of TnC might be instrumental in limiting immune cell infiltration, and in promoting astrocyte migration within the injury region, thus influencing spaciotemporal organization of the wound and the surrounding area. In fact, this study sheds new light on the role of TnC in inflammation (Jakovcevski et al., 2023; Momčilović et al., 2017).

Another interesting molecule in terms of neurite outgrowth modulation in development and regeneration is polysialic acid (PSA), best known as a posttranslational modification of the neural cell adhesion molecule (NCAM). Thiesler et al. summarize in a mini-review the functions of PSA in the modulation of NCAM, and discuss how they lead to malformations during brain development of PSA deficient mice, with a focus on how they may be linked to altered behavior in the mouse model and to the neurodevelopmental predispositions for psychiatric disorders. They further highlight the functions of PSA in interneuron development, in myelination and in microglia regulation. An interesting view on interneurons, specifically the fast-spiking parvalbumin-expressing interneurons comes from the laboratory of Andreas Faissner (Mueller-Buehl et al.) These authors studied a specialized ECM structure named perineuronal net (PNN), which envelops these neurons and regulates their synaptic input. To study the relationship between PNNs, parvalbumin interneurons, and synaptic distribution on these cells, they used a quadruple knockout mouse deficient for the ECM molecules brevican, neurocan, TnC, and tenascin-R. The disorganization of PNNs in these mice was accompanied by a reduction of inhibitory synapses and an increase of excitatory synaptic elements along the PNNs. Another study in this Research Topic examined the impact of early life stress on the PNNs around parvalbumin interneurons in rats exposed to maternal deprivation (Jakovljevic et al.) The authors’ findings indicate that PNNs enwrapping parvalbumin interneurons in the medial prefrontal cortex are adversely affected by early life stress.

Finally, Leifeld et al. reviewed the roles of the ECM, and in particular of the large ECM glycoprotein reelin, in granule cell dispersion, a morphologically prominent feature frequently observed in hippocampal tissue from patients suffering from temporal lobe epilepsy (TLE). While some studies have proposed that deficiency of the glycoprotein reelin, a protein that is well known for orchestrating neuronal layer formation in the developing cerebral cortex, contributes to the occurrence of postnatal granule cell dispersion others suggest that granule cell dispersion in the human hippocampus might represent a non-pathological morphological variation. The review discusses different interpretations of GCD related to TLE and different views on the role of the ECM protein reelin in the formation of this morphological peculiarity.

In summary, the Research Topic “*Extracellular matrix in development and regeneration of the nervous system*” has collected experimental studies and reviews focusing on the exploration of molecular mechanisms of various ECM molecules in development and function, with special attention given to structural and functional disorders of the nervous system, and their potential therapeutic relevance. We hope that this Research Topic will contribute to the understanding of the molecular mechanisms underlying the effects of various extracellular matrix components, while shedding new light on some of the present hot spots of ECM translational research, such as axonal regeneration, glial scar, the impact of perineuronal nets on synaptic plasticity.
